# Mapping the Evidence on Vitamin D, Vitamin B12, and Folate Deficiencies and Their Potential Impact on Treatment Outcomes in Patients With Cancer in Sub-Saharan Africa: Protocol for a Scoping Review

**DOI:** 10.2196/91276

**Published:** 2026-10-20

**Authors:** Olufunmilayo Olukemi Akapo, Olubunmi Margaret Ogbodu, Teke Apalata

**Affiliations:** 1 School of Pathology, Faculty of Medicine and Health Sciences, Walter Sisulu University, Sisson Street, Mthatha, Eastern Cape, South Africa, +2747 502 2204; 2 School of Biomedical Medicines, Faculty of Medicine and Health Sciences, Walter Sisulu University, Mthatha, Eastern Cape, South Africa

**Keywords:** vitamin D, vitamin B12, folate, micronutrient deficiency, cancer, chemotherapy outcomes, oncology, nutrition

## Abstract

**Background:**

Micronutrient deficiencies, particularly of vitamin D, vitamin B12, and folate, are increasingly recognized as important, yet underexplored contributors to adverse outcomes in oncology care. These micronutrients are essential for immune regulation, DNA synthesis and repair, cell proliferation, and metabolic homeostasis, all of which are frequently disrupted in patients with cancer. Deficiencies may arise due to cancer-related metabolic changes, malabsorption, anorexia, and treatment-related toxicities, potentially influencing therapeutic response, treatment tolerance, survival, and quality of life. Despite growing evidence, findings remain fragmented across cancer types, treatment modalities, and geographical settings.

**Objective:**

This scoping review aims to systematically map the available evidence on the prevalence, determinants, and reported clinical associations of vitamin D, vitamin B12, and folate deficiencies among adult patients with cancer. The review will also explore how these deficiencies are associated with treatment-related outcomes, including toxicity, therapeutic response, survival, hospitalization, and quality of life, and will identify reported screening, supplementation, and nutritional management practices in oncology care.

**Methods:**

The review protocol follows the Joanna Briggs Institute methodology for scoping reviews and will be reported according to the PRISMA-ScR (Preferred Reporting Items for Systematic Reviews and Meta-Analyses extension for Scoping Reviews). A comprehensive search of PubMed and MEDLINE, Scopus, ScienceDirect, and relevant gray literature sources will be conducted for studies published between 2000 and 2025. Eligible studies will include observational, interventional, mixed methods, and qualitative studies examining vitamin D, vitamin B12, and/or folate deficiencies in adult oncology populations. Two reviewers will independently screen studies, extract data, and chart findings, whereas a third reviewer will resolve disagreements. Extracted data will include prevalence estimates, determinants of deficiencies, clinical outcomes, and nutritional management strategies. Findings will be synthesized descriptively and presented using thematic mapping, summary tables, and evidence charts.

**Results:**

A preliminary search was conducted in May 2026. Study selection commenced on June 30, 2026, following the completion of database searches and removal of duplicate records and was finalized within 8 weeks by August 30, 2026. The anticipated timelines for each stage of the review are presented in the Results section below. Preliminary search outputs from the PubMed database demonstrate the feasibility of the proposed search strategy. The final review will report the number of studies identified, screened, included, and excluded at each stage using a PRISMA-ScR flow diagram.

**Conclusions:**

The scoping review is expected to inform future systematic reviews, guide clinical research priorities, and support the integration of nutritional assessment and monitoring into personalized oncology care, particularly in resource-limited settings.

## Introduction

### Background

Emerging evidence highlights deficiencies in vitamin D, vitamin B12, and folate as key, though often underestimated, contributors to compromised treatment outcomes in oncology [1-6]. These micronutrients are integral to immune modulation, DNA synthesis and repair, cell proliferation, and overall metabolic homeostasis functions that are especially salient in the context of cancer pathophysiology and treatment [7-10]. Vitamin D deficiency is widespread among patients with cancer, with reported rates ranging dramatically from 14% to 92% depending on cancer type and assessment criteria [1]. Similarly, vitamin B12 impairment appears to be common in oncology settings. Among hospitalized patients with cancer, alterations in B12 status have been observed, with both depletion and elevated levels linked to poor clinical status and adverse outcomes [11]. Folate deficiency, while less frequently reported in cancer-specific cohorts, is a recognized global public health concern, and its role in carcinogenesis, treatment tolerance, and prognosis cannot be dismissed [12]. Suboptimal levels of these vitamins are more than laboratory anomalies; they have been associated with tangible clinical consequences. Vitamin D deficiency has been linked to poorer prognosis in patients undergoing adjuvant chemotherapy or neoadjuvant radiotherapy, potentially through its known effects on apoptosis, angiogenesis, inflammation, and metastasis [13,14]. Vitamin B12 and folate play central roles in one-carbon metabolism, a pathway crucial for DNA methylation and repair, and deficiencies are implicated in hematologic toxicity and chemotherapy-related side effects [15,16].

Furthermore, recent epidemiological data highlight a societal surge in vitamin B deficiencies. In England, hospital admissions for nonfolate B vitamin deficiency and vitamin B12 or folate deficiency anemia increased significantly in 2023 to 2024 compared with historical figures [17,18]. Worldwide, inadequate intake of folate and related B vitamins affects a substantial portion of the population [19,20]. Beyond cancer, vitamin deficiencies have been linked to psychiatric symptoms and metabolic disturbances. For instance, combined deficiencies in vitamin D, vitamin B9 (folate), and vitamin B12 have been associated with worsened clinical severity and metabolic syndrome in psychiatric disorders, highlighting systemic implications that may also be relevant in oncological contexts [20-24]. Despite the high burden of micronutrient deficiencies in sub-Saharan Africa, evidence on micronutrient supplementation among patients with cancer in the region remains scarce. Existing African studies have primarily focused on documenting micronutrient deficiencies rather than evaluating supplementation interventions, and most oncology nutrition recommendations continue to rely on evidence generated outside Africa [25,26]. Recent evidence underscores the prevalence and potential impact of micronutrient deficiencies across diverse cancer populations, yet a comprehensive synthesis of this evidence remains lacking.

### Rationale for This Scoping Review

Although individual studies have examined vitamin deficiencies in patients with cancer, a fragmented understanding of their collective prevalence, determinants, and implications for treatment outcomes remains. However, there is sparse evidence in sub-Saharan Africa on micronutrient supplementation in oncology. This scoping review is particularly suited to mapping this heterogeneity, identifying gaps in current knowledge, and guiding future research and clinical practice in sub-Saharan Africa. It aims to systematically map the literature on vitamin D, vitamin B12, and folate deficiencies in patients with cancer, emphasizing the prevalence and determinants of these deficiencies across cancer types, stages, and treatment contexts; associations with treatment outcomes, including efficacy, toxicity, survival, and quality of life; and current practices regarding screening, supplementation, and nutritional management in oncology care. In addition, the study aims to gather existing evidence and identify knowledge gaps regarding vitamin D, vitamin B12, and folate deficiencies in oncological populations rather than developing clinical guidelines or evaluating supplementation efficacy by synthesizing current evidence and highlighting research gaps. The findings are expected to inform future focused systematic reviews, interventional studies, and context-specific research priorities.

## Methods

### Overview

The reviewers (OOA, OMO, and TA) designed the scoping review protocol in accordance with the Joanna Briggs Institute (JBI) methodological framework for scoping reviews and will report it following the PRISMA-ScR (Preferred Reporting Items for Systematic Reviews and Meta-Analyses extension for Scoping Reviews) checklist [27]. The authors chose the JBI methodology because it provides a structured framework that ensures transparency, reproducibility, and rigor in scoping reviews. It provides a comprehensive, systematic approach to synthesizing evidence, whereas the PRISMA-ScR tool is a checklist that helps authors, reviewers, and readers understand the key elements of a scoping review’s methodology and results. This approach ensures the inclusion of literature with diverse study designs, encompassing both peer-reviewed research and gray literature. The PRISMA-ScR tool supports comprehensive reporting methods and findings, helping improve the quality of scoping reviews by enhancing transparency and uniformity in reporting. The use of the JBI methodology will ensure transparency and methodological rigor. This review conforms to the revised methodology proposed by Arksey and O’Malley [28] and updated by Levac et al [29], ensuring systematic rigor through five fundamental steps: (1) identifying the research question; (2) identifying relevant studies; (3) study selection; (4) charting the data; and (5) collating, summarizing, and reporting the results.

### Formulating the Research Question

This scoping review seeks to systematically map the available evidence on vitamin D, vitamin B12, and folate deficiencies among adult patients with cancer in sub-Saharan Africa. The review aims to examine the prevalence and determinants of these deficiencies; their reported associations with treatment-related outcomes; and the screening, supplementation, and nutritional management practices described in oncology care. The following research questions were developed to guide the search strategy, study selection, data extraction, and synthesis of evidence.

The primary research question guiding this review is what evidence exists regarding vitamin D, vitamin B12, and folate deficiencies; their determinants; and reported clinical associations with adult patients with cancer in sub-Saharan Africa?

The research subquestions are as follows:

- What is the occurrence of vitamin D, vitamin B12, and folate deficiencies among adult patients with cancer in sub-Saharan Africa?
- What determinants (eg, cancer type, treatment modality, disease stage, and demographic or clinical factors) are associated with these deficiencies in sub-Saharan Africa?
- What micronutrient deficiency is associated with cancer therapy in sub-Saharan Africa?
- What screening, supplementation, or nutritional management strategies have been described in oncology settings in sub-Saharan Africa?

The study selection process will be illustrated using the PRISMA-ScR flow diagram upon completion of the review. However, a blank PRISMA-ScR flowchart (Figure 1) is provided in the manuscript to outline the intended reporting format.

Hence, the following objectives will be met in this scoping review:

- To systematically map the available evidence on the prevalence, determinants, and reported clinical associations of vitamin D, vitamin B12, and folate deficiencies among adult patients with cancer
- To explore how these deficiencies are associated with treatment-related outcomes, including toxicity, therapeutic response, survival, hospitalization, and quality of life, as well as identify reported screening, supplementation, and nutritional management practices in oncology care

**Figure 1.**
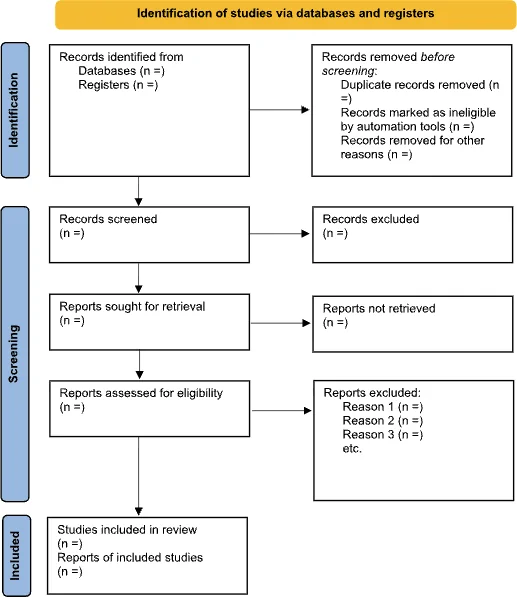
PRISMA-ScR (Preferred Reporting Items for Systematic Reviews and Meta-Analyses extension for Scoping Reviews) visually represented flowchart illustrating the article selection process for scoping reviews.

### Developing a Search Strategy

A comprehensive search of the electronic databases (PubMed, ScienceDirect, and Scopus, as well as gray literature sources) for studies published between 2000 and 2025 will be conducted. For the electronic database search, a carefully developed combination of keywords and search terms will be used to ensure the inclusion of the most relevant studies following thorough consideration by the multidisciplinary review team. The relevant keywords and synonyms or MeSH terms, such as “Neoplasms,” and Boolean operators (“OR” and “AND”) will be applied. These search terms will include study keywords and phrases such as “micronutrient supplementation in oncology” OR “Vitamin D deficiency in cancer patients” OR “vitamin B12 deficiency in adult cancer patients” OR “cancer therapy with micronutrients” AND “Sub-Saharan Africa” OR “Nigeria” OR “Uganda” OR “South Africa” OR “clinical outcomes” AND dates of publication, including other filters applied. Additional gray literature will be searched through cancer research institutes’ organizational reports and reference lists from included studies, including consulting websites such as the World Health Organization, the Institutional Repository for Information Sharing, and the United Nations. The selected articles will be compiled and managed using EndNote (version 20; Clarivate Analytics) to organize references and eliminate duplicate entries. The study selection process is expected to be finalized within 8 weeks, from June 30, 2026, to August 30, 2026, in accordance with the PRISMA-ScR checklist. The initial database search was conducted on May 25, 2026. The comprehensive PubMed search string is provided in Multimedia Appendix 1. After extracting all relevant records from the databases and removing duplicates, the Covidence platform (Veritas Health Innovation) will streamline data, facilitating collaboration among the reviewers during the screening stages [30].

### Establishing the Inclusion Criteria

Well-defined inclusion criteria are crucial for ensuring a systematic, transparent, and reproducible selection process in a scoping review. They set clear boundaries, capture diverse types of evidence, and enhance replicability, enabling future researchers to validate the findings or expand the review. In this review, studies aligned with the research question and scope will be included unless they meet explicit exclusion criteria. The research topic will include peer-reviewed original research articles on vitamin D, vitamin B12, and folate deficiency in adult patients with cancer, as well as reviews, systematic reviews, and meta-analyses. The population, concept, and context framework (Textbox 1) defines the study’s overarching inclusion and exclusion criteria. Four foundational concepts apply to this scoping review: (1) micronutrients (vitamin D, vitamin B12, folate, and relevant deficiencies), (2) health condition (cancer or malignancies), (3) outcomes (treatment-related outcomes and toxicities), and (4) geographic context (all countries in sub-Saharan Africa).

Textbox 1. **Inclusion and exclusion criteria.**

**Inclusion criteria**

- Population: adult patients with cancer across all cancer types and stages
- Geographic scope: conducted in any country in sub-Saharan Africa
- Outcomes: reports on prevalence, determinants, or clinical associations of micronutrient deficiencies (including insufficiencies) with oncology-related outcomes (eg, toxicity, response, survival, and quality of life)
- Scope of interventions: describes screening, nutritional monitoring, supplementation practices, or supportive nutritional management in oncology settings
- Study design: peer-reviewed published literature (observational, interventional, mixed methods, and qualitative studies) and secondary literature (systematic reviews and meta-analyses)
- Sources: databases and gray literature searches (including World Health Organization and national repository websites)
- Time frame and language: published between 2000 and 2026 and written in English

**Exclusion criteria**

- Alternative focus: studies focusing primarily on micronutrients other than vitamin D, vitamin B12, or folate
- Nonhuman data: animal studies, laboratory-based studies, and in vitro investigations
- Studies exclusively evaluating supplementation efficacy without reporting baseline deficiency status or clinical associations
- Irrelevant population: studies unrelated to oncology populations or conducted outside sub-Saharan Africa
- Conference abstracts lacking sufficient methodological detail
- Language barrier: non–English-language publications

Eligible studies will be published from 2000 to 2026 to provide a comprehensive mapping of evidence from the last 2 decades and synthesize current publicly available information on the subject. From the early 1970s onward, vitamins A and D gained recognition for their anticancer properties, and since the 2000s, deficiencies in vitamin D, folate, and vitamin B12 have been tied to prognosis, chemotherapy tolerance, and survival outcomes. Information on cancer stage, treatment phase (pretreatment, active treatment, survivorship, or palliative care), and treatment modality will be extracted to provide clinical context for interpreting micronutrient status and associated outcomes when reported.

### Study Time Frame

The selection of the study period from 2000 to 2026 represents the period during which the World Health Organization and the Food and Agriculture Organization established a link between chronic noncommunicable diseases, including cancer, and micronutrient deficiencies, as well as infectious diseases [31]. This discovery made it justifiable to integrate micronutrient therapies into frameworks for cancer prevention and supportive therapy, especially among groups with dietary deficiencies. By the year 2000, micronutrient deficiencies had been documented in cancer initiation, progression, and treatment outcomes. Micronutrients’ essential roles in DNA repair, regulation of cell proliferation, immunological function, and antioxidant defense mechanisms lend them biological validity.

### Data Extraction

A standardized data extraction form will be developed and piloted to ensure clarity and consistency. The form will capture key details to ensure effectiveness. No universal diagnostic cutoff values will be imposed during study selection because definitions of vitamin D, vitamin B12, and folate deficiency vary across clinical guidelines, laboratory methods, and study settings. Instead, the review will extract and report the threshold criteria used in each included study. These definitions will be charted during data extraction to facilitate transparent comparison and identify inconsistencies across the literature. The study characteristics will include author, year of publication, title of the study, aim of the study, study design, study and country setting, study population, age group, intervention characteristics, study outcomes, key findings, and recommendations from the study. The review will include studies reporting both micronutrient deficiency and insufficiency provided that these classifications are clearly defined by the original study authors. Due to variability in international guidelines and laboratory thresholds, particularly for vitamin D and vitamin B12, the review will not impose uniform diagnostic cutoff values. Instead, the specific definitions and threshold criteria used in each included study will be extracted and reported to support transparent comparison and interpretation of findings across studies. The data charting form is shown in Textbox 2.

Textbox 2. **Data charting form.**

- Author and year of publication
- Country and study setting
- Study design and methodology
- Cancer type and study population characteristics
- Micronutrient assessed (vitamin D, vitamin B12, and/or folate)
- Definition or diagnostic criteria used for deficiency
- Reported prevalence and determinants of deficiency
- Treatment context (chemotherapy, radiotherapy, surgery, palliative care, etc)
- Reported clinical associations, including treatment toxicity, treatment response, survival outcomes, quality of life, hospitalization, or disease progression
- Study outcomes (cancer-related mortality, quality of life, treatment response, and progression of the disease)
- Screening or nutritional assessment practices reported
- Recommendations from the study
- Supplementation or nutritional management strategies described
- Key findings and identified research gaps
- Diagnostic criteria and threshold values used to define vitamin D, vitamin B12, or folate deficiency or insufficiency

Quality assessment is not mandatory for scoping reviews; we will conduct a descriptive quality appraisal using the JBI critical appraisal checklists for relevant study designs to provide context to the evidence. Two reviewers (OOA and OMO) will independently extract data from each study using a standardized form to ensure transparency. The extraction process will take place in 2 stages: an initial title and abstract screening phase in which both reviewers independently collect data and a full-text screening phase in which both reviewers assess completeness and correctness. Any disagreement will be resolved through discussion and consensus, with a third reviewer (TA) serving as tiebreaker. This structured approach enhances the rigor and reliability of data extraction, ensuring consistent capture of key information related to the research questions. The inclusion and exclusion criteria for the initial data extraction of this scoping review were assessed using a random sample of citations to ensure consistency. Upon finalization, the interrater reliability of another random sample of identified studies will be evaluated using the Cohen κ coefficient. The Cohen κ coefficient provides a data-driven means to validate these reviews, helping researchers and practitioners pinpoint inconsistencies and biases before the results are skewed. This leads to improved trust in the results and enhanced credibility of the review outcomes [32]. Sampling will continue until a κ score of 0.85 is achieved. To maintain a high level of agreement, interrater reliability will be routinely assessed throughout the title and abstract screening process. Beyond the Cohen κ coefficient, discrepancies will be resolved through discussion and consensus among the 3 reviewers (OOA, OMO, and TA).

### Data Assessment, Summarization, and Presentation of the Results

A descriptive numerical summary will outline the number of studies included; their publication years, study designs, and cancer types; and the geographical and clinical settings represented. Tables and charts will illustrate the distribution of evidence across vitamin D, vitamin B12, and folate deficiencies in relation to cancer treatment outcomes. This review will primarily examine reported associations between vitamin D, vitamin B12, and folate deficiencies and oncology-related clinical outcomes rather than establishing causality. Accordingly, the review will focus on outcomes commonly reported in the literature, including associations between micronutrient deficiencies and overall survival or disease progression, treatment-related toxicity, and tolerance to chemotherapy or radiotherapy; treatment response and hospitalization outcomes; patient-reported quality of life and functional status; and prevalence and determinants of micronutrient deficiencies across cancer populations and treatment contexts. Secondary outcomes will include reported screening approaches, nutritional assessment practices, supplementation strategies, and supportive nutritional management practices within oncology care settings.

The framework by Thomas and Harden [33] for thematic synthesis will then be applied to organize the findings around the main and sub–research questions. The main question and subquestions will guide the coding of the descriptive themes that will be obtained from the consulted literature. The qualitative data analysis software ATLAS.ti (ATLAS.ti Scientific Software Development GmbH) will be used to manage emergent codes and themes.

### Ethical Considerations

As this review will use data from published studies, no ethics approval is required.

## Results

### Overview

The study does not have any funding. A preliminary search was conducted in May 2026. The literature searches were conducted up to the point of manuscript submission in January 2026, and all studies retrieved by that date were included. The study selection process was finalized within 8 weeks, from June 30, 2026, to August 30, 2026. This scoping review will map the existing scientific literature on current oncology research to highlight, among other things, the impact of deficiencies in vitamin D, vitamin B12, and folate on cancer treatment outcomes and support the integration of nutritional monitoring into personalized cancer care, particularly in resource-constrained settings such as the sub-Saharan region. The data analysis has not started yet, with preliminary results expected to be finalized by December 2027. We anticipate that the results will be submitted for publication later in 2027.

The anticipated timelines for each stage are shown in Table 1.

Preliminary search results from the PubMed database are shown in Multimedia Appendix 1.

**Table 1.** Anticipated study timelines.

Activity	Timelines
Identifying relevant studies	June 30, 2026-July 14, 2026
Deduplication	July 15, 2026-July 21, 2026
Study selection	July 22, 2026- August 30, 2026
Charting the data	September 1, 2026 – October 25, 2026
Data analysis	October 26, 2026 - November 25, 2026
Collating, summarizing, and reporting the results	November 25, 2026 – December 29, 2026

### Patient and Public Involvement

No patients or members of the public were involved in establishing the research questions, developing the methods, interpreting the results, or contributing to the dissemination plans for this scoping review protocol.

## Discussion

### Principal Findings

This scoping review is expected to provide a comprehensive mapping of the available evidence on vitamin D, vitamin B12, and folate deficiencies among patients with cancer in sub-Saharan Africa. Specifically, the review will identify reported prevalence estimates; determinants of micronutrient deficiencies; and their documented associations with treatment-related outcomes, including toxicity, treatment response, survival, hospitalization, and quality of life. The review will also summarize existing screening approaches, supplementation practices, and nutritional management strategies used in oncology care while highlighting evidence gaps requiring further investigation.

### Comparison to Prior Work

The available evidence on vitamin D, vitamin B12, and folate deficiencies and their potential impact on treatment outcomes in patients with cancer in sub-Saharan Africa will be mapped. Although deficiencies in these micronutrients are well documented in the general population, their implications in oncology remain underexplored and fragmented across different cancer types, treatment modalities, and geographical settings.

### Strengths and Limitations

By systematically synthesizing the existing literature, this review will highlight current knowledge; identify gaps; and clarify the extent to which these deficiencies influence clinical outcomes, including treatment response, toxicity, survival, and quality of life. Several limitations are anticipated in this scoping review.

First, as a scoping review, the objective is to map and summarize the available evidence rather than conduct a critical appraisal or meta-analysis of study quality. The protocol is limited to studies published in English between 2000 and 2026, which may introduce language and publication bias by excluding relevant evidence in other languages or from earlier periods. Second, heterogeneity in study designs, populations, diagnostic criteria for deficiencies, and outcome measures may complicate direct comparisons and synthesis. Third, reliance on published literature and selected gray literature sources may lead to omission of unpublished data, conference abstracts, or region-specific reports, particularly from low-resource settings. Finally, the review will focus primarily on studies conducted in sub-Saharan Africa, which, while providing valuable contextual insights, may limit the generalizability of the findings to other regions. Despite these limitations, the review is expected to provide an important overview of the current evidence landscape regarding micronutrient deficiencies in oncology populations across sub-Saharan Africa. The findings will help identify research priorities and inform the design of future systematic reviews and intervention studies in this area. The anticipated findings may also guide clinicians in implementing personalized nutritional monitoring and supplementation protocols to improve treatment tolerance, treatment efficacy, and quality of life for patients with cancer.

### Future Directions

In summary, the intersection of micronutrient deficiencies and cancer care represents a clinically relevant yet underinvestigated domain. As precision oncology advances, nutritional optimization, including correction of vitamin D, vitamin B12, and folate deficiencies, has the potential to become a cost-effective, accessible adjunct to conventional cancer therapies.

### Dissemination Plans

Findings will be disseminated through a peer-reviewed publication in a high-impact oncology and nutrition journal, conference presentations, and stakeholder briefings to inform oncology nutrition policy and practices.

## Supplementary material

10.2196/91276 Multimedia Appendix 1 PubMed search strategy.

10.2196/91276 Checklist 1 PRISMA-ScR checklist.

## Abbreviations

JBI: Joanna Briggs Institute
PRISMA-ScR: Preferred Reporting Items for Systematic Reviews and Meta-Analyses extension for Scoping Reviews

## References

[1] Afonso ML, Capelas ML, Pimenta NM (2025). A systematic review of vitamin D supplementation in oncology: chance of science or effectiveness?. Nutrients.

[2] Minisola S, Ferrone F, Danese V (2019). Controversies surrounding vitamin D: focus on supplementation and cancer. Int J Environ Res Public Health.

[3] Shahvegharasl Z, Pirouzpanah S, Mahboob SA (2020). Effects of cholecalciferol supplementation on serum angiogenic biomarkers in breast cancer patients treated with tamoxifen: a controlled randomized clinical trial. Nutrition.

[4] Tan SY, Tsoukalas T, Javier K, Fazon T, Singh S, Vardy J (2026). Recommendations on the surveillance and supplementation of vitamins and minerals for upper gastrointestinal cancer survivors: a scoping review. J Cancer Surviv.

[5] Forma A, Grunwald A, Zembala P (2024). Micronutrient status and breast cancer: a narrative review. Int J Mol Sci.

[6] Zwart NR, Franken MD, Tissing WJ (2023). Folate, folic acid, and chemotherapy-induced toxicities: a systematic literature review. Crit Rev Oncol Hematol.

[7] Gombart AF, Pierre A, Maggini S (2020). A review of micronutrients and the immune system-working in harmony to reduce the risk of infection. Nutrients.

[8] Fenech MF, Bull CF, van Klinken BJ (2023). Protective effects of micronutrient supplements, phytochemicals and phytochemical-rich beverages and foods against DNA damage in humans: a systematic review of randomized controlled trials and prospective studies. Adv Nutr.

[9] Ames BN (2018). Prolonging healthy aging: longevity vitamins and proteins. Proc Natl Acad Sci U S A.

[10] Olukemi Akapo O, Ogbodu OM (2025). A scoping review protocol of the efficacy and safety of high-dose intravenous vitamin C in the treatment of advanced solid tumors in African populations. F1000Res.

[11] Urbanski G, Hamel JF, Prouveur B (2020). Strength of the association of elevated vitamin B12 and solid cancers: an adjusted case-control study. J Clin Med.

[12] Bo Y, Zhu Y, Tao Y (2020). Association between folate and health outcomes: an umbrella review of meta-analyses. Front Public Health.

[13] Bao Y, Li Y, Gong Y, Huang Q, Cai S, Peng J (2020). Vitamin D status and survival in stage II-III colorectal cancer. Front Oncol.

[14] Yu X, Liu B, Zhang N, Wang Q, Cheng G (2021). Immune response: a missed opportunity between vitamin D and radiotherapy. Front Cell Dev Biol.

[15] Saraei P, Ghasemi M, Talebi A, Vafaeinezhad A, Saberzadeh J (2025). Nutritional strategies in oncology: a narrative review of advances in folate-targeted therapeutic approaches for cancer treatment. Nutr Cancer.

[16] Wei D, Wang L, Zuo X, Bresalier RS (2021). Vitamin D: promises on the horizon and challenges ahead for fighting pancreatic cancer. Cancers (Basel).

[17] Reeves K, Watson SI, Crothers H (2025). Prevalence of malnutrition among hospital admissions to English NHS hospitals over two economically constrained decades: evidence from routine health data. BMJ Public Health.

[18] Stratton RJ (2025). Managing malnutrition and multimorbidity in primary care: dietary approaches to reduce treatment burden. Proc Nutr Soc.

[19] Naderi N, House JD (2018). Recent developments in folate nutrition. Adv Food Nutr Res.

[20] Faugere M, Maakaron É, Achour V (2025). Vitamin D, B9, and B12 deficiencies as key drivers of clinical severity and metabolic comorbidities in major psychiatric disorders. Nutrients.

[21] Tobin D, Vuckovic A, Sarris J (2024). Targeting divergent pathways in the nutritional management of depression. Nutrients.

[22] Kaviani M, Nikooyeh B, Etesam F (2022). Effects of vitamin D supplementation on depression and some selected pro-inflammatory biomarkers: a double-blind randomized clinical trial. BMC Psychiatry.

[23] Jahan-Mihan A, Stevens P, Medero-Alfonso S (2024). The role of water-soluble vitamins and vitamin D in prevention and treatment of depression and seasonal affective disorder in adults. Nutrients.

[24] Amrein K, Scherkl M, Hoffmann M (2020). Vitamin D deficiency 2.0: an update on the current status worldwide. Eur J Clin Nutr.

[25] Najah Q, Selim R, Almosilhy NA (2025). Prevalence of undernutrition and its contributing factors among cancer patients in Africa: a systematic review and meta-analysis of observational studies. Ann Nutr Metab.

[26] Chauhan A, Khedkar RD (2025). Nutritional challenges in cancer patients: impact, deficiencies, and management strategies. BIO Web Conf.

[27] Hadie SN (2024). ABC of a scoping review: a simplified JBI scoping review guideline. Educ Med J.

[28] Arksey H, O’Malley L (2005). Scoping studies: towards a methodological framework. Int J Soc Res Methodol.

[29] Levac D, Colquhoun H, O’Brien KK (2010). Scoping studies: advancing the methodology. Implement Sci.

[30] Covidence.

[31] (2002). Human vitamin and mineral requirements: report of a joint FAO/WHO expert consultation. https://www.fao.org/4/y2809e/y2809e00.htm.

[32] McHugh ML (2012). Interrater reliability: the kappa statistic. Biochem Med (Zagreb).

[33] Thomas J, Harden A (2008). Methods for the thematic synthesis of qualitative research in systematic reviews. BMC Med Res Methodol.

