# Supplementary material for: Mapping the Evidence on Vitamin D, Vitamin B12, and Folate Deficiencies and Their Potential Impact on Treatment Outcomes in Patients With Cancer in Sub-Saharan Africa: Protocol for a Scoping Review
Source: JMIR Res Protoc. 2026 Oct 20;15:e91276. doi: 10.2196/91276 (PMC13660966; doi:10.2196/91276)
Supplement: Supplementary file 2 [file resprot-v15-e91276-s001.docx]

**Precise PubMed search string**

Main research question: What evidence exists regarding vitamin D, vitamin B12, and folate deficiencies and their reported associations with treatment-related outcomes in adult cancer patients in sub-Saharan Africa?

| #1  Filters applied: 01 Jan 2000 – 01 Jan 2026 | (("Neoplasms"[MeSH Terms] OR ("cancer s"[All Fields] OR "cancerated"[All Fields] OR "canceration"[All Fields] OR "cancerization"[All Fields] OR "cancerized"[All Fields] OR "cancerous"[All Fields] OR "Neoplasms"[MeSH Terms] OR "Neoplasms"[All Fields] OR "cancer"[All Fields] OR "cancers"[All Fields]) OR "adult"[All Fields] OR "patient"[All Fields]) AND ("vitamin d"[MeSH Terms] OR "Vitamin B 12"[MeSH Terms] OR "Folic Acid"[MeSH Terms] OR "vitamin d"[All Fields] OR "vitamin B12"[All Fields] OR ("Folic Acid"[Supplementary Concept] OR "Folic Acid"[All Fields] OR "folate"[All Fields] OR "Folic Acid"[MeSH Terms] OR ("folic"[All Fields] AND "acid"[All Fields]) OR "folates"[All Fields]) OR "Folic Acid"[All Fields] OR ("deficiences"[All Fields] OR "deficiencies"[All Fields] OR "deficiency"[MeSH Subheading] OR "deficiency"[All Fields] OR "deficient"[All Fields] OR "deficients"[All Fields])) AND ("treatment outcome"[MeSH Terms] OR "mortality"[MeSH Terms] OR ("mortality"[MeSH Subheading] OR "mortality"[All Fields] OR "survival"[All Fields] OR "survival"[MeSH Terms] OR "survivability"[All Fields] OR "survivable"[All Fields] OR "survivals"[All Fields] OR "survive"[All Fields] OR "survived"[All Fields] OR "survives"[All Fields] OR "surviving"[All Fields]) OR ("toxic"[All Fields] OR "toxical"[All Fields] OR "toxically"[All Fields] OR "toxicant"[All Fields] OR "toxicant s"[All Fields] OR "toxicants"[All Fields] OR "toxicated"[All Fields] OR "toxication"[All Fields] OR "toxicities"[All Fields] OR "toxicity"[MeSH Subheading] OR "toxicity"[All Fields] OR "toxicity s"[All Fields] OR "toxics"[All Fields]) OR "treatment-related outcomes"[All Fields]) AND ("Sub-Saharan Africa"[All Fields] OR "South Africa"[All Fields] OR "Kenya"[All Fields] OR "Nigeria"[All Fields] OR "Uganda"[All Fields] OR "Ghana"[All Fields])) AND (2000/1/1:2026/1/1[pdat]) | 580 results |
| --- | --- | --- |
| # 2  Filters applied: Meta-analysis, Randomized Clinical Trial, Review, English, from 01 Jan 2000 – 01 Jan 2026 | (("Neoplasms"[MeSH Terms] OR ("cancer s"[All Fields] OR "cancerated"[All Fields] OR "canceration"[All Fields] OR "cancerization"[All Fields] OR "cancerized"[All Fields] OR "cancerous"[All Fields] OR "Neoplasms"[MeSH Terms] OR "Neoplasms"[All Fields] OR "cancer"[All Fields] OR "cancers"[All Fields]) OR "adult"[All Fields] OR "patient"[All Fields]) AND ("vitamin d"[MeSH Terms] OR "Vitamin B 12"[MeSH Terms] OR "Folic Acid"[MeSH Terms] OR "vitamin d"[All Fields] OR "vitamin B12"[All Fields] OR ("Folic Acid"[Supplementary Concept] OR "Folic Acid"[All Fields] OR "folate"[All Fields] OR "Folic Acid"[MeSH Terms] OR ("folic"[All Fields] AND "acid"[All Fields]) OR "folates"[All Fields]) OR "Folic Acid"[All Fields] OR ("deficiences"[All Fields] OR "deficiencies"[All Fields] OR "deficiency"[MeSH Subheading] OR "deficiency"[All Fields] OR "deficient"[All Fields] OR "deficients"[All Fields])) AND ("treatment outcome"[MeSH Terms] OR "mortality"[MeSH Terms] OR ("mortality"[MeSH Subheading] OR "mortality"[All Fields] OR "survival"[All Fields] OR "survival"[MeSH Terms] OR "survivability"[All Fields] OR "survivable"[All Fields] OR "survivals"[All Fields] OR "survive"[All Fields] OR "survived"[All Fields] OR "survives"[All Fields] OR "surviving"[All Fields]) OR ("toxic"[All Fields] OR "toxical"[All Fields] OR "toxically"[All Fields] OR "toxicant"[All Fields] OR "toxicant s"[All Fields] OR "toxicants"[All Fields] OR "toxicated"[All Fields] OR "toxication"[All Fields] OR "toxicities"[All Fields] OR "toxicity"[MeSH Subheading] OR "toxicity"[All Fields] OR "toxicity s"[All Fields] OR "toxics"[All Fields]) OR "treatment-related outcomes"[All Fields]) AND ("Sub-Saharan Africa"[All Fields] OR "South Africa"[All Fields] OR "Kenya"[All Fields] OR "Nigeria"[All Fields] OR "Uganda"[All Fields] OR "Ghana"[All Fields])) AND ((meta-analysis[Filter] OR randomizedcontrolledtrial[Filter] OR review[Filter]) AND (2000/1/1:2026/1/1[pdat]) AND (english[Filter])) | 134 results |
| #3  Filters applied: Meta-analysis, Randomized Clinical Trial, Review, Systematic Review, English, Humans  from 01 Jan 2000 – 01 Jan 2026 | (("Neoplasms"[MeSH Terms] OR ("cancer s"[All Fields] OR "cancerated"[All Fields] OR "canceration"[All Fields] OR "cancerization"[All Fields] OR "cancerized"[All Fields] OR "cancerous"[All Fields] OR "Neoplasms"[MeSH Terms] OR "Neoplasms"[All Fields] OR "cancer"[All Fields] OR "cancers"[All Fields]) OR "adult"[All Fields] OR "patient"[All Fields]) AND ("vitamin d"[MeSH Terms] OR "Vitamin B 12"[MeSH Terms] OR "Folic Acid"[MeSH Terms] OR "vitamin d"[All Fields] OR "vitamin B12"[All Fields] OR ("Folic Acid"[Supplementary Concept] OR "Folic Acid"[All Fields] OR "folate"[All Fields] OR "Folic Acid"[MeSH Terms] OR ("folic"[All Fields] AND "acid"[All Fields]) OR "folates"[All Fields]) OR "Folic Acid"[All Fields] OR ("deficiences"[All Fields] OR "deficiencies"[All Fields] OR "deficiency"[MeSH Subheading] OR "deficiency"[All Fields] OR "deficient"[All Fields] OR "deficients"[All Fields])) AND ("treatment outcome"[MeSH Terms] OR "mortality"[MeSH Terms] OR ("mortality"[MeSH Subheading] OR "mortality"[All Fields] OR "survival"[All Fields] OR "survival"[MeSH Terms] OR "survivability"[All Fields] OR "survivable"[All Fields] OR "survivals"[All Fields] OR "survive"[All Fields] OR "survived"[All Fields] OR "survives"[All Fields] OR "surviving"[All Fields]) OR ("toxic"[All Fields] OR "toxical"[All Fields] OR "toxically"[All Fields] OR "toxicant"[All Fields] OR "toxicant s"[All Fields] OR "toxicants"[All Fields] OR "toxicated"[All Fields] OR "toxication"[All Fields] OR "toxicities"[All Fields] OR "toxicity"[MeSH Subheading] OR "toxicity"[All Fields] OR "toxicity s"[All Fields] OR "toxics"[All Fields]) OR "treatment-related outcomes"[All Fields]) AND ("Sub-Saharan Africa"[All Fields] OR "South Africa"[All Fields] OR "Kenya"[All Fields] OR "Nigeria"[All Fields] OR "Uganda"[All Fields] OR "Ghana"[All Fields])) AND ((meta-analysis[Filter] OR randomizedcontrolledtrial[Filter] OR review[Filter]) AND (humans[Filter]) AND (2000/1/1:2026/1/1[pdat]) AND (english[Filter])) | 116 results |
| #4  Filters applied: From 2000/1/1 to 2026/1/1, Meta-Analysis, Randomized Controlled Trial, Review, Systematic review, English, Humans, Adult: 19+ years. | (("Neoplasms"[MeSH Terms] OR ("cancer s"[All Fields] OR "cancerated"[All Fields] OR "canceration"[All Fields] OR "cancerization"[All Fields] OR "cancerized"[All Fields] OR "cancerous"[All Fields] OR "Neoplasms"[MeSH Terms] OR "Neoplasms"[All Fields] OR "cancer"[All Fields] OR "cancers"[All Fields]) OR "adult"[All Fields] OR "patient"[All Fields]) AND ("vitamin d"[MeSH Terms] OR "Vitamin B 12"[MeSH Terms] OR "Folic Acid"[MeSH Terms] OR "vitamin d"[All Fields] OR "vitamin B12"[All Fields] OR ("Folic Acid"[Supplementary Concept] OR "Folic Acid"[All Fields] OR "folate"[All Fields] OR "Folic Acid"[MeSH Terms] OR ("folic"[All Fields] AND "acid"[All Fields]) OR "folates"[All Fields]) OR "Folic Acid"[All Fields] OR ("deficiences"[All Fields] OR "deficiencies"[All Fields] OR "deficiency"[MeSH Subheading] OR "deficiency"[All Fields] OR "deficient"[All Fields] OR "deficients"[All Fields])) AND ("treatment outcome"[MeSH Terms] OR "mortality"[MeSH Terms] OR ("mortality"[MeSH Subheading] OR "mortality"[All Fields] OR "survival"[All Fields] OR "survival"[MeSH Terms] OR "survivability"[All Fields] OR "survivable"[All Fields] OR "survivals"[All Fields] OR "survive"[All Fields] OR "survived"[All Fields] OR "survives"[All Fields] OR "surviving"[All Fields]) OR ("toxic"[All Fields] OR "toxical"[All Fields] OR "toxically"[All Fields] OR "toxicant"[All Fields] OR "toxicant s"[All Fields] OR "toxicants"[All Fields] OR "toxicated"[All Fields] OR "toxication"[All Fields] OR "toxicities"[All Fields] OR "toxicity"[MeSH Subheading] OR "toxicity"[All Fields] OR "toxicity s"[All Fields] OR "toxics"[All Fields]) OR "treatment-related outcomes"[All Fields]) AND ("Sub-Saharan Africa"[All Fields] OR "South Africa"[All Fields] OR "Kenya"[All Fields] OR "Nigeria"[All Fields] OR "Uganda"[All Fields] OR "Ghana"[All Fields]) AND (("meta analysis"[Publication Type] OR "randomized controlled trial"[Publication Type] OR "review"[Publication Type]) AND "humans"[MeSH Terms] AND 2000/01/01:2026/01/01[Date - Publication] AND "english"[Language])) AND ((meta-analysis[Filter] OR randomizedcontrolledtrial[Filter] OR review[Filter]) AND (humans[Filter]) AND (2000/1/1:2026/1/1[pdat]) AND (english[Filter]) AND (alladult[Filter])) | 67 results |
| #5  Filters applied: Abstract, Free full text, Meta-Analysis, Randomized Controlled Trial, Review, Systematic Review, English, Humans, from 2000/1/1 - 2026/1/1 | (("Neoplasms"[MeSH Terms] OR ("cancer s"[All Fields] OR "cancerated"[All Fields] OR "canceration"[All Fields] OR "cancerization"[All Fields] OR "cancerized"[All Fields] OR "cancerous"[All Fields] OR "Neoplasms"[MeSH Terms] OR "Neoplasms"[All Fields] OR "cancer"[All Fields] OR "cancers"[All Fields]) OR "adult"[All Fields] OR "patient"[All Fields]) AND ("vitamin d"[MeSH Terms] OR "Vitamin B 12"[MeSH Terms] OR "Folic Acid"[MeSH Terms] OR "vitamin d"[All Fields] OR "vitamin B12"[All Fields] OR ("Folic Acid"[Supplementary Concept] OR "Folic Acid"[All Fields] OR "folate"[All Fields] OR "Folic Acid"[MeSH Terms] OR ("folic"[All Fields] AND "acid"[All Fields]) OR "folates"[All Fields]) OR "Folic Acid"[All Fields] OR ("deficiences"[All Fields] OR "deficiencies"[All Fields] OR "deficiency"[MeSH Subheading] OR "deficiency"[All Fields] OR "deficient"[All Fields] OR "deficients"[All Fields])) AND ("treatment outcome"[MeSH Terms] OR "mortality"[MeSH Terms] OR ("mortality"[MeSH Subheading] OR "mortality"[All Fields] OR "survival"[All Fields] OR "survival"[MeSH Terms] OR "survivability"[All Fields] OR "survivable"[All Fields] OR "survivals"[All Fields] OR "survive"[All Fields] OR "survived"[All Fields] OR "survives"[All Fields] OR "surviving"[All Fields]) OR ("toxic"[All Fields] OR "toxical"[All Fields] OR "toxically"[All Fields] OR "toxicant"[All Fields] OR "toxicant s"[All Fields] OR "toxicants"[All Fields] OR "toxicated"[All Fields] OR "toxication"[All Fields] OR "toxicities"[All Fields] OR "toxicity"[MeSH Subheading] OR "toxicity"[All Fields] OR "toxicity s"[All Fields] OR "toxics"[All Fields]) OR "treatment-related outcomes"[All Fields]) AND ("Sub-Saharan Africa"[All Fields] OR "South Africa"[All Fields] OR "Kenya"[All Fields] OR "Nigeria"[All Fields] OR "Uganda"[All Fields] OR "Ghana"[All Fields]) AND (("meta analysis"[Publication Type] OR "randomized controlled trial"[Publication Type] OR "review"[Publication Type]) AND "humans"[MeSH Terms] AND 2000/01/01:2026/01/01[Date - Publication] AND "english"[Language]) AND (("meta analysis"[Publication Type] OR "randomized controlled trial"[Publication Type] OR "review"[Publication Type]) AND "humans"[MeSH Terms] AND 2000/01/01:2026/01/01[Date - Publication] AND "english"[Language] AND "adult"[MeSH Terms])) AND ((ffrft[Filter]) AND (fha[Filter]) AND (meta-analysis[Filter] OR randomizedcontrolledtrial[Filter] OR review[Filter] OR systematicreview[Filter]) AND (humans[Filter]) AND (2000/1/1:2026/1/1[pdat]) AND (english[Filter])) | 38 results |
